# A computational classification method of breast cancer images using the VGGNet model

**DOI:** 10.3389/fncom.2022.1001803

**Published:** 2022-11-04

**Authors:** Abdullah Khan, Asfandyar Khan, Muneeb Ullah, Muhammad Mansoor Alam, Javed Iqbal Bangash, Mazliham Mohd Suud

**Affiliations:** ^1^Institute of Computer Science and Information Technology, ICS/IT FMCS the University of Agriculture, Peshawar, Pakistan; ^2^Faculty of Computing, Riphah International University, Islamabad, Pakistan; ^3^Faculty of Computing and Informatics, Multimedia University, Cyberjaya, Malaysia

**Keywords:** breast cancer, VGGNet, CNN, decision tree, KNN, LeNet

## Abstract

Cancer is one of the most prevalent diseases worldwide. The most prevalent condition in women when aberrant cells develop out of control is breast cancer. Breast cancer detection and classification are exceedingly difficult tasks. As a result, several computational techniques, including k-nearest neighbor (KNN), support vector machine (SVM), multilayer perceptron (MLP), decision tree (DT), and genetic algorithms, have been applied in the current computing world for the diagnosis and classification of breast cancer. However, each method has its own limitations to how accurately it can be utilized. A novel convolutional neural network (CNN) model based on the Visual Geometry Group network (VGGNet) was also suggested in this study. The 16 layers in the current VGGNet-16 model lead to overfitting on the training and test data. We, thus, propose the VGGNet-12 model for breast cancer classification. The VGGNet-16 model has the problem of overfitting the breast cancer classification dataset. Based on the overfitting issues in the existing model, this research reduced the number of different layers in the VGGNet-16 model to solve the overfitting problem in this model. Because various models of the VGGNet, such as VGGNet-13 and VGGNet-19, were developed, this study proposed a new version of the VGGNet model, that is, the VGGNet-12 model. The performance of this model is checked using the breast cancer dataset, as compared to the CNN and LeNet models. From the simulation result, it can be seen that the proposed VGGNet-12 model enhances the simulation result as compared to the model used in this study. Overall, the experimental findings indicate that the suggested VGGNet-12 model did well in classifying breast cancer in terms of several characteristics.

## Introduction

Breast cancer is the second most common disease in the world and is the leading cause of death from cancer in women between the ages of 20 and 39. Breast cancer comprises 23% of all cancer diagnoses, and 14% of these cases result in death (Parkin et al., 2001). Considering that more women die from breast cancer each year than from any other type of cancer, it is the most prevalent disease that predominantly kills women globally (Asri et al., 2016). The uncontrolled growth of aberrant cells is a symptom of this illness (Solbjør, 2008). In Asia, Europe, and the USA, the lifetime risk of developing breast cancer in women is around 1/40, 1/12, and 1/8, respectively. Breast cancer was the cause of 502,000 fatalities in 2005 (Plevritis et al., 2018).

The most effective way to stop the spread of breast cancer is through early detection. A reliable and effective system of detection is necessary to detect breast cancer at an early stage. Medical image processing, digital pathology, magnetic resonance imaging (MRI), computed tomography (CT) scan, ultrasound, and nuclear imaging are some of the breast cancer detection techniques that have been developed (Sahan et al., 2007; Spanhol et al., 2016, 2017; Dabeer et al., 2019). Histopathology, which includes a biopsy of the breast tissue of the affected parts of the breast, captures breast cancer pictures. The pathologist removes the breast tissues that are impacted by the tumor and uses haematoxylin and eosin (H&E) to stain them. The damaged tissues of the tumor are then examined under a microscope to look for cancer cells with malignant characteristics in their cellular structure. The collection of all the microscopic pictures that show how the tumor has affected a patient is utilized to create a computerized detection system. Manual detection is tedious, extremely challenging, and likely to be compromised by human mistakes. Finding out if a tumor is benign or malignant is the major goal of the detection system. To further prevent cancer and minimize its spread, malignant tumor should be treated as soon as feasible. Numerous machine algorithms must be used to determine whether a tumor is benign or malignant, which includes a binary classification problem (Dabeer et al., 2019). It has been demonstrated in the past that a machine learning algorithm outperforms a human pathologist. Numerous earlier studies demonstrated that processing medical images using different machine learning algorithms yields more accurate results compared to having a human pathologist diagnose the images. A study by Phillips in Europe demonstrated that a collection of machine learning algorithms with breast cancer images produces results with better detection conclusions (Aswathy and Jagannath, 2017). For the diagnosis, prognosis, and categorization of cancer, He et al. (2016) used a variety of machine learning classifiers, including artificial neural networks (ANNs), SVM, and k-nearest neighbor (KNN). Similarly, Jiang et al. (2019) employed a variety of machine learning approaches to determine if a tumor was malignant or benign, including SVMs, probabilistic neural networks, and KNN with a signal-to-noise ratio and principal component analysis (PCA). Additionally, Cruz and Wishart (2006) employed several strategies for categorizing breast cancer (BC), including a decision tree with and without a feature selection strategy. Dongale et al. (2015), in their research, employed the genetic algorithm and support vector machine (SVM) as two examples of machine learning methods. The KNN model was employed in another study (Lavanya and Rani, 2011) to classify breast cancer. Additionally, J48 and multilayer perceptron (MLP) data mining techniques were suggested by a previous study (Vanneschi et al., 2011) for the prediction of breast cancer. To compare the effectiveness of several deep learning models, including CNN and AlexNet, using BreaKH breast cancer data, Medjahed et al. (2013) offered various deep learning models for the BreaKH cancer classification. Saabith et al. (2014) created an AGGNet-based CNN model for the classification of breast cancer. The primary goal of the study was to evaluate the effectiveness of the new CNN powered by AGGNet and determine with accuracy whether a breast tumor is benign or malignant. Albarqouni et al. (2016) employed a CNN approach based on deep learning to classify breast cancer pictures. Similarly, Araújo et al. (2017) proposed deep learning models for breast cancer classification to check the performance of the proposed deep learning models. This study used the opinion of an expert pathologist for the purpose of comparison between an expert pathologist and a deep learning model. The simulation results of the study showed that deep learning performs better than an expert pathologist. The study also supports the idea that high-resolution pictures and improved algorithms will enhance the effectiveness and precision of cancer diagnosis. Based on the diagnosis of cancer, which is carried out with the help of MRI pictures, a vast amount of medical image data is transformed. The high dimensionality of the data makes it challenging to distinguish between malignant and non-cancerous pictures. Deep learning techniques are required since they can distinguish between normal and abnormal photographs with accuracy. Deep learning is more dependable since it automatically categorizes cancer photographs. At present, it is challenging for deep learning to carry out the automatic classification of pathological breast cancer images based on convolutional neural networks (CNNs) for the following reasons: The number of CNN parameters increases rapidly due to a continuous deepening of the model, which easily leads to overfitting of the model. A large number of breast cancer histopathological images are used as training data for training CNN to reduce the risk of overfitting. However, the cost of obtaining a large number of labeled breast cancer images is expensive. Therefore, in case of limited breast cancer image data, we need to reduce the model's overfitting risk from the perspective of reducing CNN parameters and using data augmentation methods. The 16 layers in the current VGG-16 model led to overfitting of the training and test data. The previous models are improved in this study, which also decreases the number of layers to 12—6 convolutional, 3 Max pooling, 1 flattening, and 2 fully connected layers. In terms of several characteristics, including accuracy, loss, recall, precision, f-measure, specificity, and sensitivity, the performance of the suggested model, that is, upgraded VGGNet, was evaluated. Therefore, to increase the accuracy of breast cancer picture classification, this study suggests a unique VGGNET-12-based CNN. The following are key contributions of the current study:

1. We proposed an efficient deep learning VGGNet-12-based CNN model for breast cancer image classification.
2. The proposed model enhances the performance of the existing VGGsNet, which causes the overfitting problem.
3. The proposed model reduces the convolutional layers to 12 layers, which solve the problem of overfitting.

The remaining study is structured as follows: Section Related work addresses the associated works in the article. Similar to Section Materials and methods, Section Proposed VGGNet architecture design based on CNN delves into the proposed works' techniques. Section Experimental results and discussion covers the findings and analysis of the VGGNet method in comparison to other algorithms. Section Conclusion provides the conclusion of the study.

## Related work

Several literature studies demonstrated that machine-learning algorithms outperformed human pathologists in the analysis of medical pictures and provided findings that were more accurate than those produced by human pathologists. In this section, we discuss some of the greatest and most effective learning techniques that shed light on prior improvements that many researchers had advised regarding how to improve the learning effectiveness of their networks in order to acquire some positive and encouraging outcomes for the category.

A variety of machine learning classifiers for cancer classification, prediction, and diagnosis were used in He et al. (2016). Several experiments were carried out utilizing machine learning models such as ANNs, SVM, and KNN. According to the experiment, the ANN outperformed the SVM and KNN models in terms of accuracy. Similarly, Jiang et al. (2019) employed a variety of machine learning methods including SVMs, probabilistic neural networks, and KNN with a signal-to-noise ratio and PCA. The major focus of this study was the classification of a tumor as benign or malignant. All of these models' effectiveness is evaluated in terms of accuracy. According to the simulation results, SVM with a single-to-noise ratio and PCA had an accuracy rate of 96.33%, which showed better results than the compared models. Furthermore, Cruz and Wishart (2006) used different approaches for breast cancer (BC) classification, such as a decision tree with a feature selection approach and also one without a feature selection approach. This research also used different pre-processing and feature selection techniques to enhance the classification accuracy of breast cancer. Different analyses were conducted on three types of breast cancer. From the experimental analysis, it was concluded that the feature selection approaches could lead to high accuracy. The simulation result shows that the decision tree with the feature selection method performed well and obtained a 96.33% accuracy, which is better than the decision tree without the feature selection approach.

Dongale et al. (2015) studied and employed a few machine-learning techniques, such as the genetic algorithm and support vector machine (SVM). The effectiveness of these models was evaluated in terms of their accuracy in detecting breast cancer. The simulation results were analyzed, and it is shown that the genetic algorithm achieved a noticeably greater accuracy than the SVM while automatically choosing the optimal feature. The KNN approach was employed (Lavanya and Rani, 2011) for the categorization of breast cancer. To test the effectiveness of the model used to analyse the performance of various values and various distances, the author of this study employed two distinct types of distance parameters. The University of Wisconsin Hospital “Breast Cancer Wisconsin Database (BCWD)” is the dataset used for the categorization goal. On the basis of the results, the KNN method was used with two different types of Manhattan and Euclidean distances. These two lengths are efficient but take more time in terms of performance and categorization. The best results are obtained for the Euclidean distance (98.70%) and Manhattan distance (98.48%) with k = 1.

Additionally, Vanneschi et al. (2011) suggested J48 and MLP data mining techniques for breast cancer prediction. They compared the effectiveness of employing J48 and MLP with and without feature selection methodologies. Under the feature selection approach, several tests were run by altering the values of the training and testing datasets. To get more accuracy and minimize loss while classifying breast cancer, the feature selection technique is the most reliable way. Medjahed et al. (2013) suggested several deep learning models for the BreaKH cancer classification to test the effectiveness of various deep learning models like AlexNet and CNN using BreaKH breast cancer data. The total simulation result demonstrates that the AlexNet model outperforms the straightforward CNN model. The AlexNet model outperformed the base CNN with an accuracy rate of 96%. Saabith et al. (2014) created an AGGNet-based CNN model for classifying breast cancer. The major goal of this study was to evaluate the effectiveness of the new CNN powered by AGGNet and to determine with accuracy whether a breast tumor is benign or malignant. This approach's effectiveness contrasted with that of the current CNN model. The simulation result, showed that the proposed AGGNet model achieved a higher accuracy than CNN. Albarqouni et al. (2016) employed a CNN approach based on deep learning to classify breast cancer pictures. The suggested CNN method, based on deep learning, performs similar to SVM. The compression of both models was carried out using 80% training and 20% testing sets of breast cancer picture data. The experimental findings demonstrate that the suggested CNN model, which is deep learning-based, achieved more accuracy than SVM. Similar to a previous study (Araújo et al., 2017), deep learning models for breast cancer categorization were also suggested in order to evaluate the effectiveness of the suggested deep learning models. To compare the results, this study employed a pathologist's professional judgement. The simulation findings demonstrate that deep learning outperforms a skilled pathologist. In a previous study, Rodrigues et al. attempted (Rodrigues Filho and Cortes, 2022) to use the VGG-7 CNN, a more straightforward model, to classify breast cancer in histopathology pictures. The results indicate that VGG-7 outperforms VGG-16 and VGG-19, with accuracy, precision, recall, and F1 scores, respectively.

In this study, Yan et al. (2021) suggested a brand-new, more sophisticated fusion network for classifying benign and aggressive breast cancer using multimodal data. They suggested a method to extract richer multilevel feature representations of the diseased picture from many convolutional layers in order to make the integration of pathological images with structured electronic medical records (EMR) data more effective. Instead of converting high-dimensional picture data to low-dimensional picture data prior to data fusion, this study employed an autoencoder to enhance the low-dimensional structured EMR data to high-dimensional type in order to reduce the information loss for each modality. Additionally, this approach is readily generalized by the autoencoder to produce good predictions using partially missing structured EMR data.

Furthermore, Khan et al. (2022) proposed a “MultiNet” architecture aimed to give rapid and precise diagnoses for breast cancer using binary classification (benign and malignant) and multiclass classification (benign, *in situ*, invasive, and normal). The proposed approach employs three well-known pre-trained models, namely DenseNet-201, NasNetMobile, and VGG16, to extract features from microscope pictures. To create a strong hybrid model, the extracted features are subsequently supplied into the concatenated layer. Using the suggested framework, two classes may be classified with an overall accuracy of 99%. Additionally, it successfully categorizes four classes with an accuracy rate of 98%. The “MultiNet” framework can be used as a diagnostic model in clinics and the medical field, thanks to these encouraging results.

## Materials and methods

The basic operations of convolutional neural networks are discussed in the following subsections.

### Convolutional neural networks

The CNN is a modified version of the basic ANN, which consists of a multilayer feed forward network. It was built specifically to process large-scale image data that are presented as multiple arrays by choosing the local and global stationary properties (Araújo et al., 2017). Similar to a multilayer perceptron (MLP), a CNN model has a variable number of layers, with each layer's neurons coupled to the next layer's by a set of trainable weights and biases (Litjens et al., 2017). The primary distinction between the CNN model and the MLP is that the CNN model captures various features through the process of convolution, resulting in input and output feature maps for each layer. The basic CNN model consists of different operations such as convolution, non-linearity, pooling, and fully connected layers (LeCun et al., 2015). The three main types of layers used in the architecture of CNN are as follows.

#### Convolutional layer

The convolutional layer is the first layer of the CNN model. Filters or kernels that operate as feature extractors are present in this layer. CNN's filters, or kernels, learn the feature representations of their input pictures and have a tiny field that spans the whole depth of the input component (Romero et al., 2015). Each neuron in the convolutional layers is grouped into feature maps that represent the input portion of the picture and is coupled to another neuron in the preceding layer through a set of learnable weights (Araújo et al., 2017). To generate new feature data, the input data are convolved with the trainable weights. A non-linear activation function is applied to the newly generated convolved feature. Every neuron in a given feature map has a weight value that is equally constrained. Varied feature maps inside the same convolutional layer have different weight values, allowing many features to be retrieved at each location (Schmidhuber, 2015). By multiplying the dot product between the entrances of the kernel and the input, each filter is convolved across the height and the breadth of the input component during the forward pass, creating a two-dimensional (2D) activation map of the associated kernel (O'Shea and Nash, 2015). A Mathematically Convolutional Layer can be represented in Equation (1).


(1) $$\begin{array}{l} Y=f\left(\sum \left(X_{{}^{*}}w_{k}\right)\right), \end{array}$$

where *X* stands for the input image, *wk* stands for the convolutional filter associated with the kth feature map, and *f*(.) stands for the non-linear activation function. In this context, the multiplication sign refers to the 2D convolutional operator, which is used to calculate the inner product of the filter model at each location of the input image. Additionally, rectified linear units (Nair and Hinton, 2010) have become quite popular (Araújo et al., 2017).

#### Pooling layer

The pooling layer is another crucial layer. It gradually reduces the size of the picture, as well as the number of parameters, the amount of computation, the network's memory footprint, and fitting control. A pooling layer is introduced between convolutional layers. Max pooling is the most popular type of pooling. By using maximum pooling for each subregion, the input image is separated into a collection of separate frames. By operating impulsively on each input depth slice, the pooling layer resizes the input picture spatially. The most typical method involves pooling layer samples with stride 2 on down side and a filter size of 22. The depth measurement does not change (Yu et al., 2014).


(2) $$\begin{array}{l} X_{kij}=\underset{pq\in R}{\max}T_{kpq}, \end{array}$$

Where the output of the pooling operation, associated with the kth feature map, is denoted by*X*_*kij*_, *T*_*kpq*_ denotes the element at location (p, q) contained by the pooling region *Ri* j, which embodies a receptive field around the position (i, j) (O'Shea and Nash, 2015).

#### Fully connected layer

Finally, after a number of max pooling and convolutional layers, the fully connected layer and the highest analysis in CNN are completed. For every activation in the preceding layer, linkages between neurons are present in this layer. A CNN architecture known as the fully connected layer consists of an input layer, a hidden layer, and an output layer. A CNN classifier is used in this layer (Romero et al., 2015). For the two consecutive layers, all nodes from the previous layer must be fully linked to every node in the subsequent levels. In the fully connected layer, the two repeated layers connect it through the weighted matrix defined as *w*^(*k*)^ ∈ *R*^*m* *k*−*l*)^ × *m*^(*k*)^. Bias term also includes the fully connected layer *b*^(*k*)^ ∈*R*^*m*(*k*)^. *O*^(*k*)^denotes the output of the fully connected layer.


(3) $$\begin{array}{l} O^{\left(k\right)}=\;{\left(l^{k-1}\right)}^{T}w^{\left(k\right)}+b^{\left(k\right)} \end{array}$$

### Data collection

The most crucial stage of any research project is data collection. Data for this study were gathered from a previous study (Cruz-Roa et al., 2014) and comprise 162 slide photographs that have been 40 × scanned. Since the slide pictures were initially exceedingly huge, a total of 277,524 patches were removed to make the data more manageable. These were separated into negative and positive categories. For instance, 78,786 of the 198,738 photographs were positive instances, meaning that they showed breast cancer, while the remaining images were negative instances that did not. However, the most prevalent kind of breast cancer, invasive ductal carcinoma, was used in this study. The dataset was created by Cruz-Roa et al. (2014). The author collected data from 162 patients and scanned them at 40 × . Table 1 shows the average malignant and benign cancer images.

**Table 1:** Distribution of images.

**Class**	**Malignant**	**Benign**
40x	85	75

**Figure 1:**
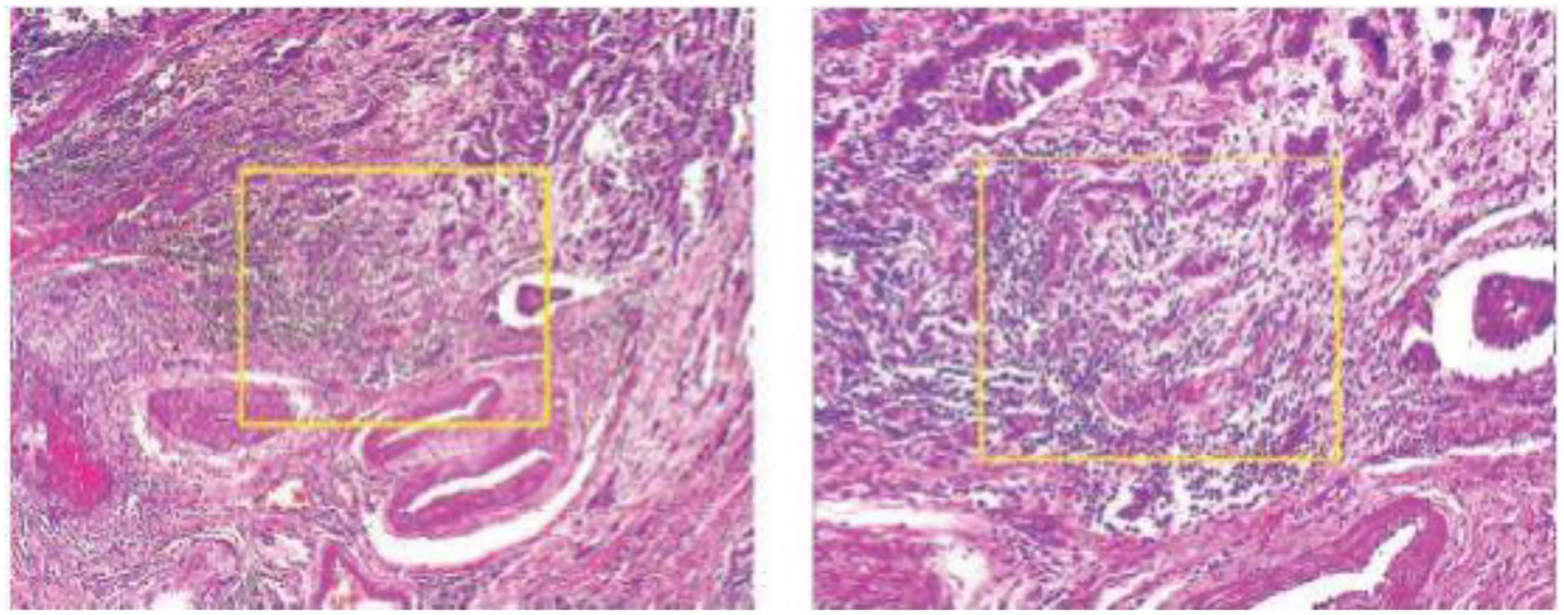
Malignant and benign tumors.

The photographs were taken using a BX-50 Olympus at a magnification of 40 × . To reduce complexity and information loss during processing, the photographs were kept without any normalization or color standards. Figure 1 displays the photographs in PNG format with an 8-bit depth and three channels.

### Image processing

Image processing techniques are applied to the breast cancer (BC) image dataset to improve the quality of images and produce the best results.

### Data augmentation

This research performed geometric transformation on the BC dataset, which kept the original feature while changing the pixel position.

### Feature detection

Feature detection is a technique of image processing. It is used to extract information from images and determine if the pixels of the image fit the feature or not.

### Sliding window crop

**Figure 2:**
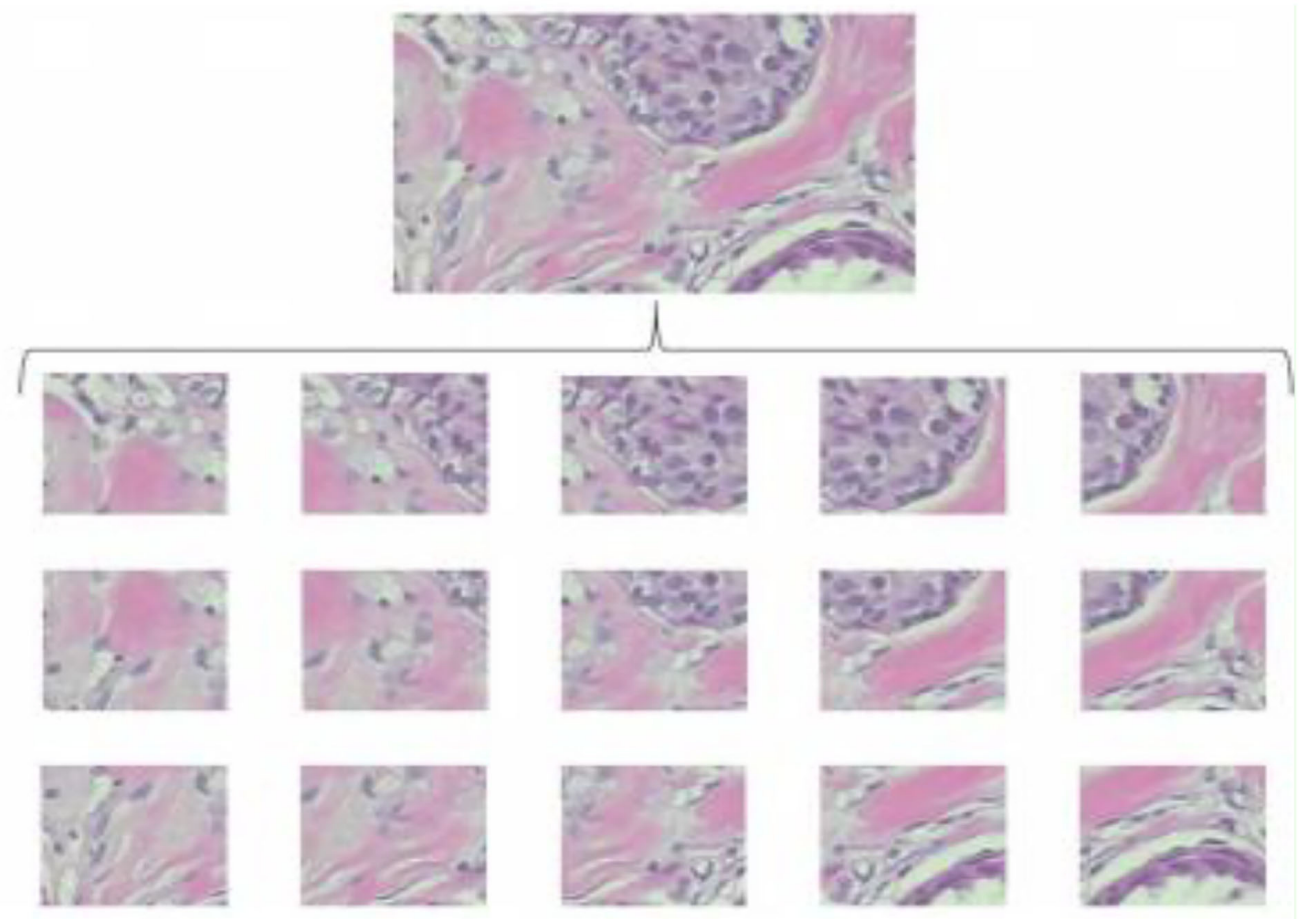
Examples of sliding window crops.

Another image processing method that is frequently used in deep learning is the sliding window crop. The deep learning algorithm architecture will get more complex if a high-resolution picture dataset is treated using a deep learning model on a large high-resolution image size. The deep learning model often includes more layers and parameters, which significantly increase complexity. In this case, deep neural network (DNN) algorithm training and testing might be exceedingly time-consuming and expensive. The first method is to crop the photographs using a sliding window. The picture step of 0.05N and the cropping of the photographs as seen in Figure 2 was used to determine the window slide size N × N. Overlays between crops in images are performed to prevent damaging the structure data too much. The total number of entire crops is specified by the following formula:


(4) $$\begin{array}{l} \left(crop\right)=2\times \frac{IMGWIDTH}{N}\times 2\times \frac{IMGWIDTH}{N} \end{array}$$

### Random crop

**Figure 3:**
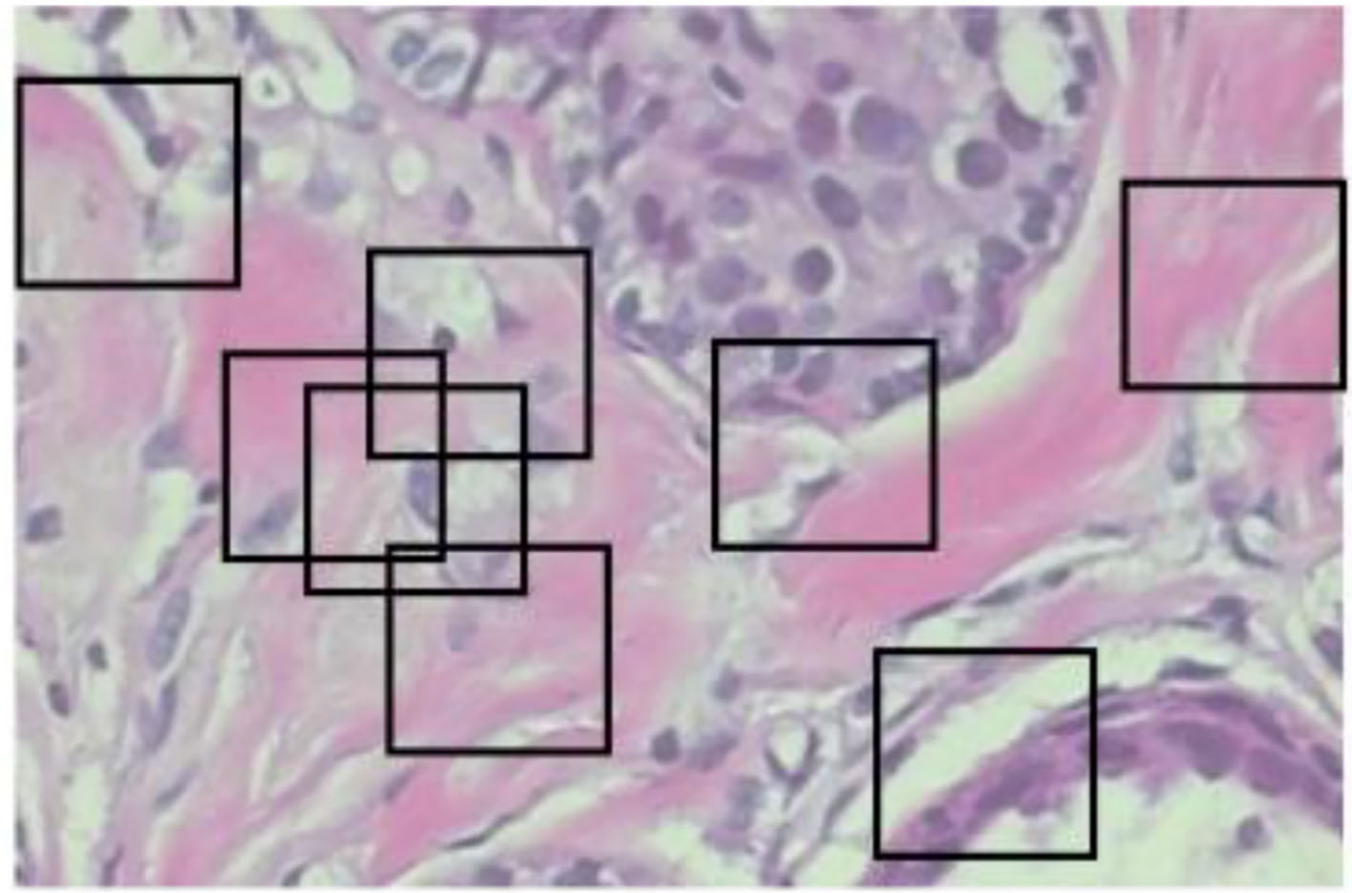
Examples of random crops.

The random crop is a second image processing technique to solve oversized problems. Instead of sliding, we set a size of (N × N) to do a random crop, as shown in Figure 3. The number of entire crops is dynamic. There will be no limit on how to crop random selectors. For benign tumor images, there will be no problem, but for malignant images, we cannot be sure if a tumor exists in every crop. Crop mined from malignant tumor images may comprise no tumor and should be classified as benign.

### Resizing

**Figure 4:**
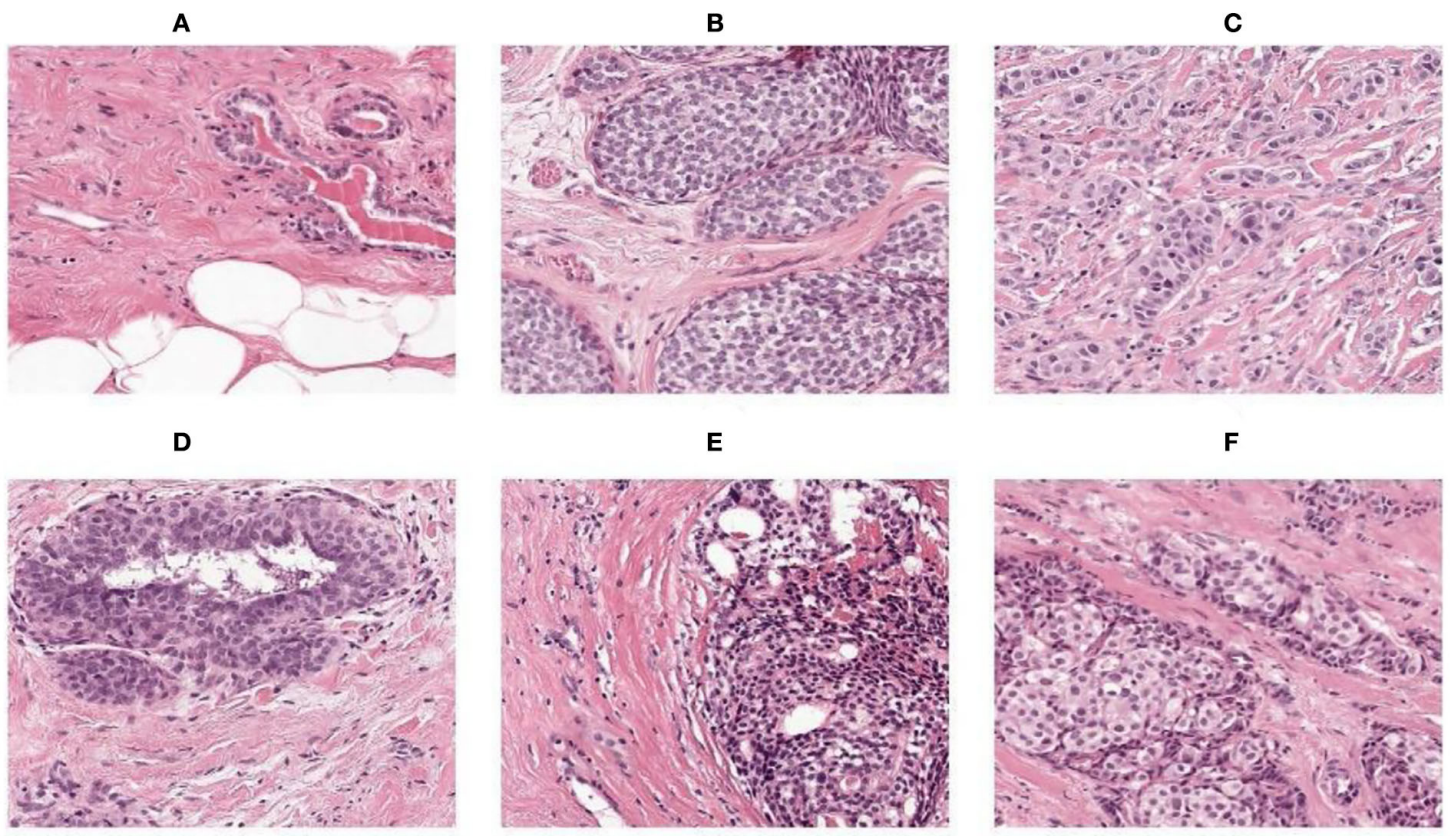
**(A–F)** Resize images.

For resizing, there exists a method of shrinking normal and abnormal images. We resized the image to the used pixel area ratio. This is the top image interpolation procedure for resizing the image. It tends to give clear normal and abnormal images and make them high resolution. Figures 4A–F shows the 50 × 50 resized images.

### Whitening

**Figure 5:**
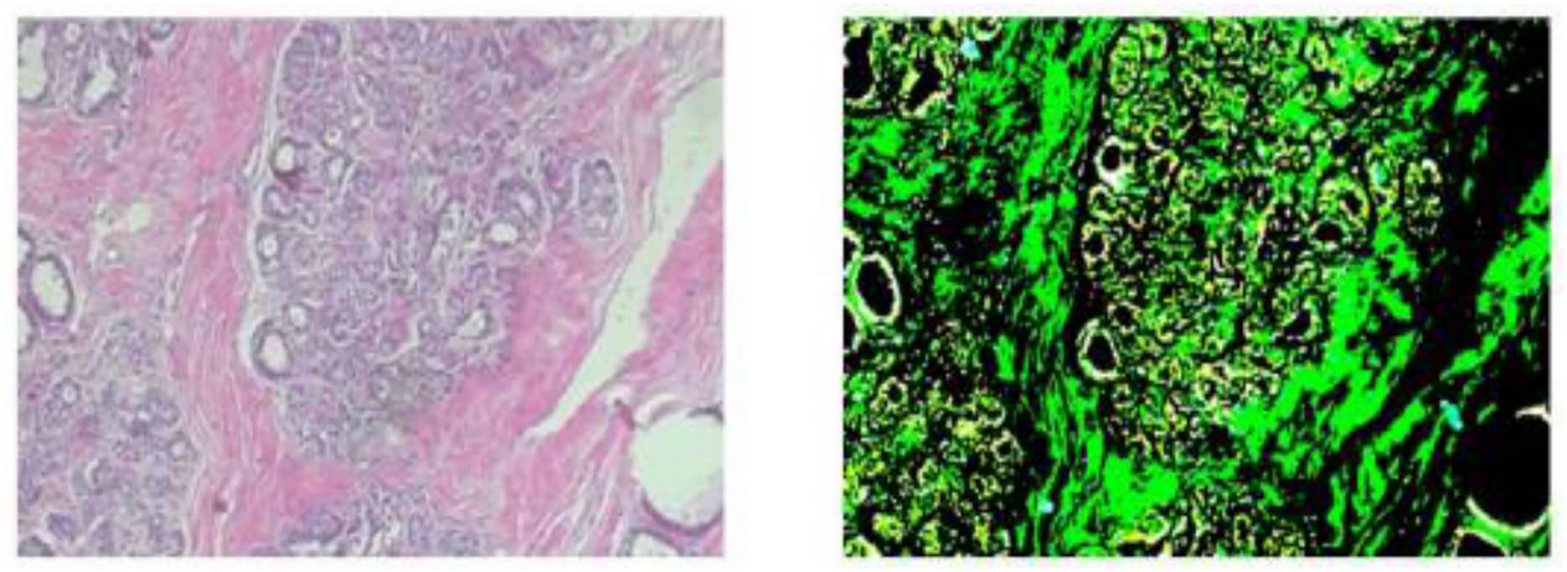
Before and after whitening.

Whitening is the pre-processing procedure for machine learning models. It is used to remove noise and extra information from images. After whitening, the images have two properties where one image has the same feature and is less correlated. This will improve the training process. Figure 5 shows images before and after whitening. First, we gave the input dataset, such as { *x*1, …………..*xm* }. Then, the matrix of x is determined as follows:


(5) $$\begin{array}{l} \begin{array}{l} \sum^{\text{​}}=\frac{1}{m}\sum_{m}^{i}x_{i}x_{i}^{t} \end{array} \end{array}$$

Therefore, we have


(6) $$\begin{array}{l} x_{not}=\;u^{t}x, \end{array}$$

where u is the vector of ∑.

This process x maps into a space that correlation eliminates; then, we have


(7) $$\begin{array}{l} x_{PCAwhite}=\frac{x_{rot}}{\sqrt{\partial }}, \end{array}$$

which is a normalized dataset.

### Row major order

**Figure 6:**
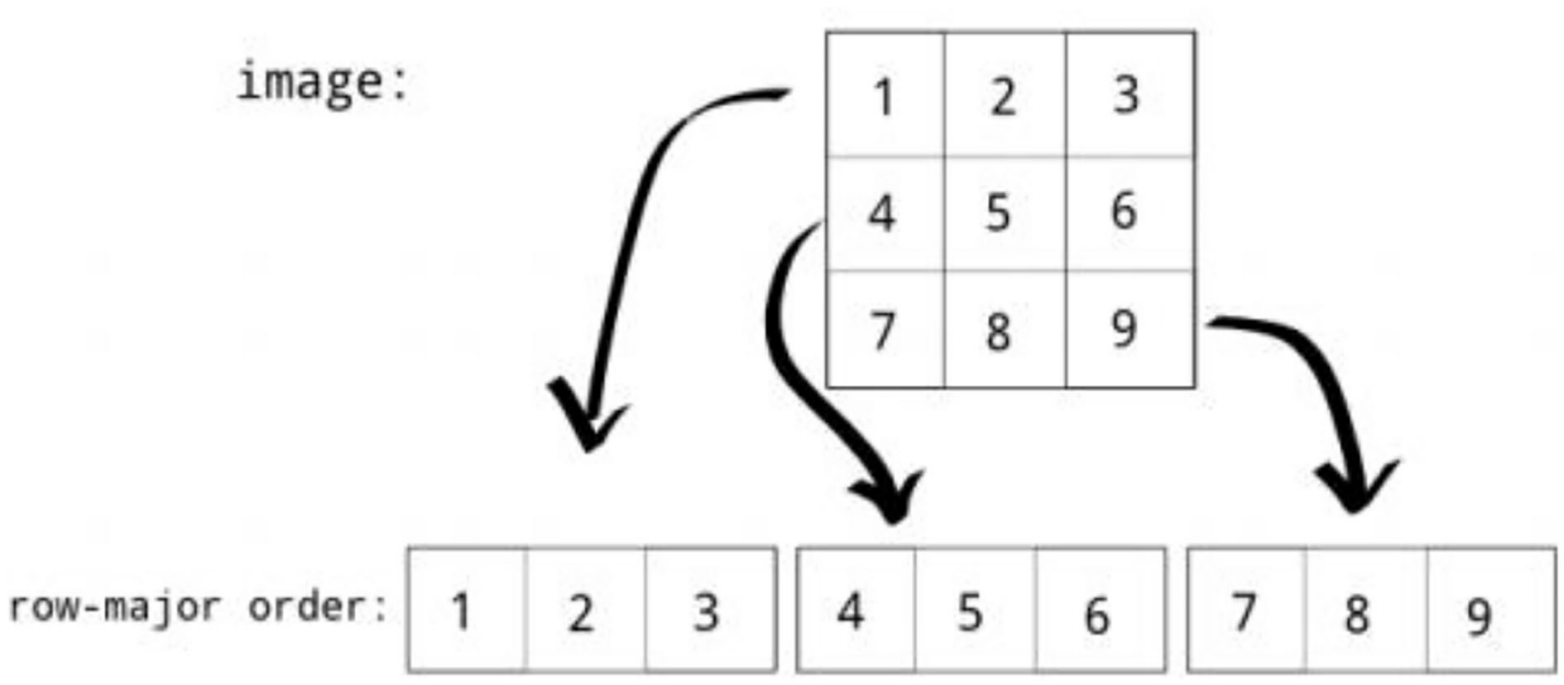
Row major order.

**Figure 7:**
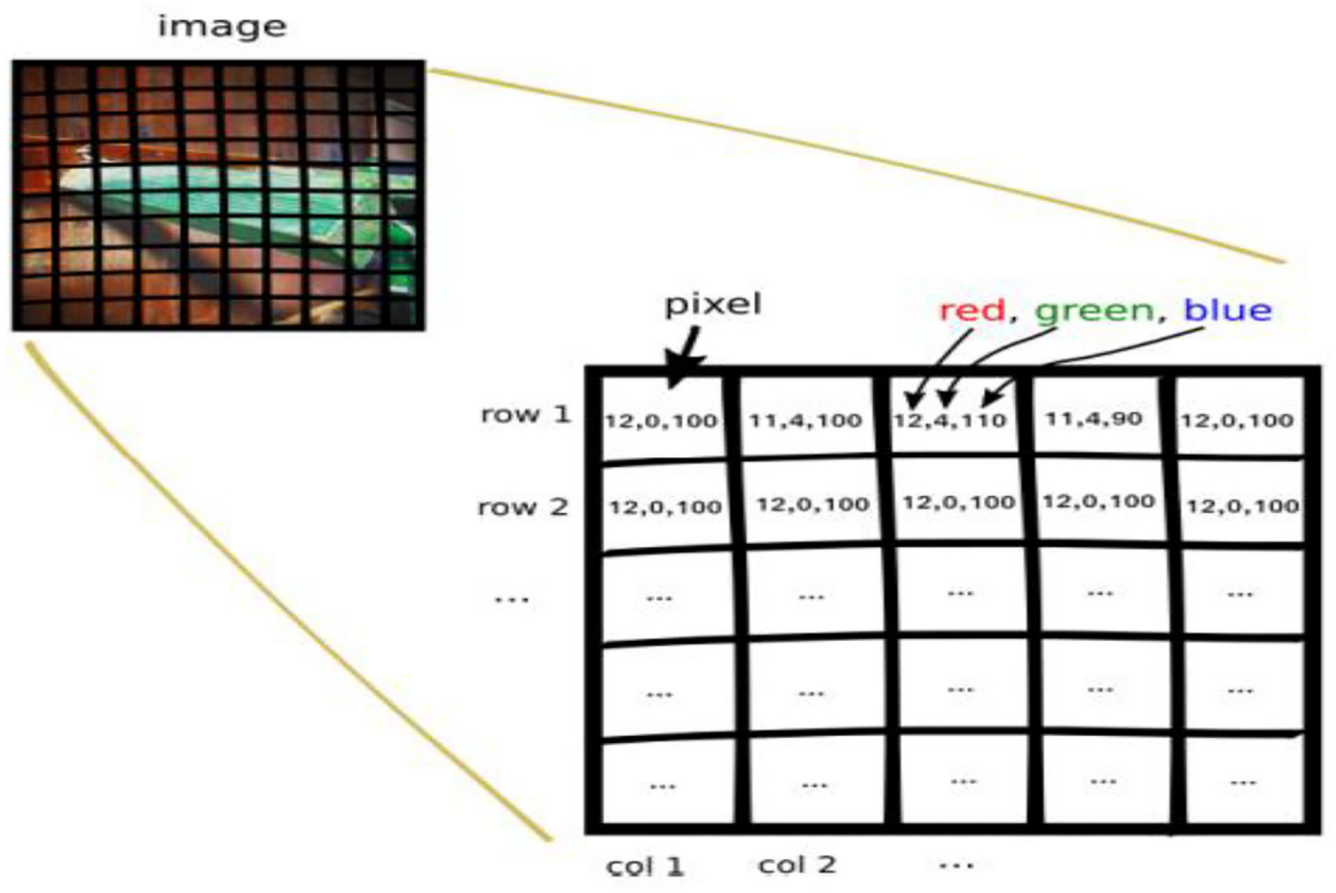
Red green blue (RGB) image.

Row major order stores two-dimensional (2D) image structures into one-dimensional (1D) structures. The grayscale image of a size of 3 by 3 pixels were organized into a structure of one dimension, as shown in Figure 6. Row major order stores longer arrays. Deep neural network (DDN) autoencoder of a red green blue (RGB) image contains pixels of red, green, and blue values, as shown in Figure 7.

### Class labeling

**Figure 8:**
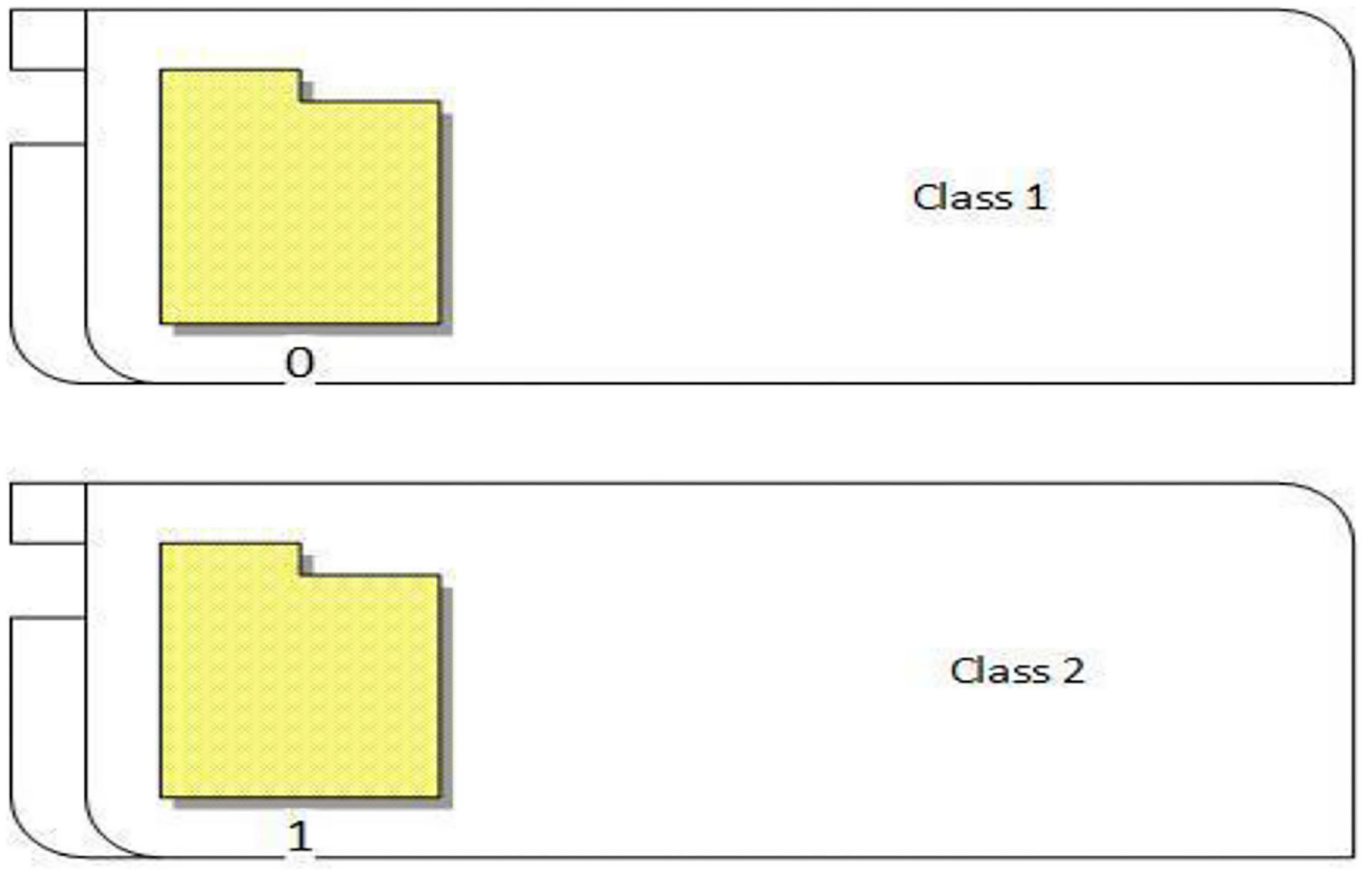
Class labeling.

The proposed approach classified the breast cancer dataset into two classes (0 and 1). As shown in Figure 8, 0 represents normal images that have no breast cancer, while 1 represents abnormal images that have breast cancer, as shown in Figure 8.

## Proposed VGGNet architecture design based on CNN

**Figure 9:**
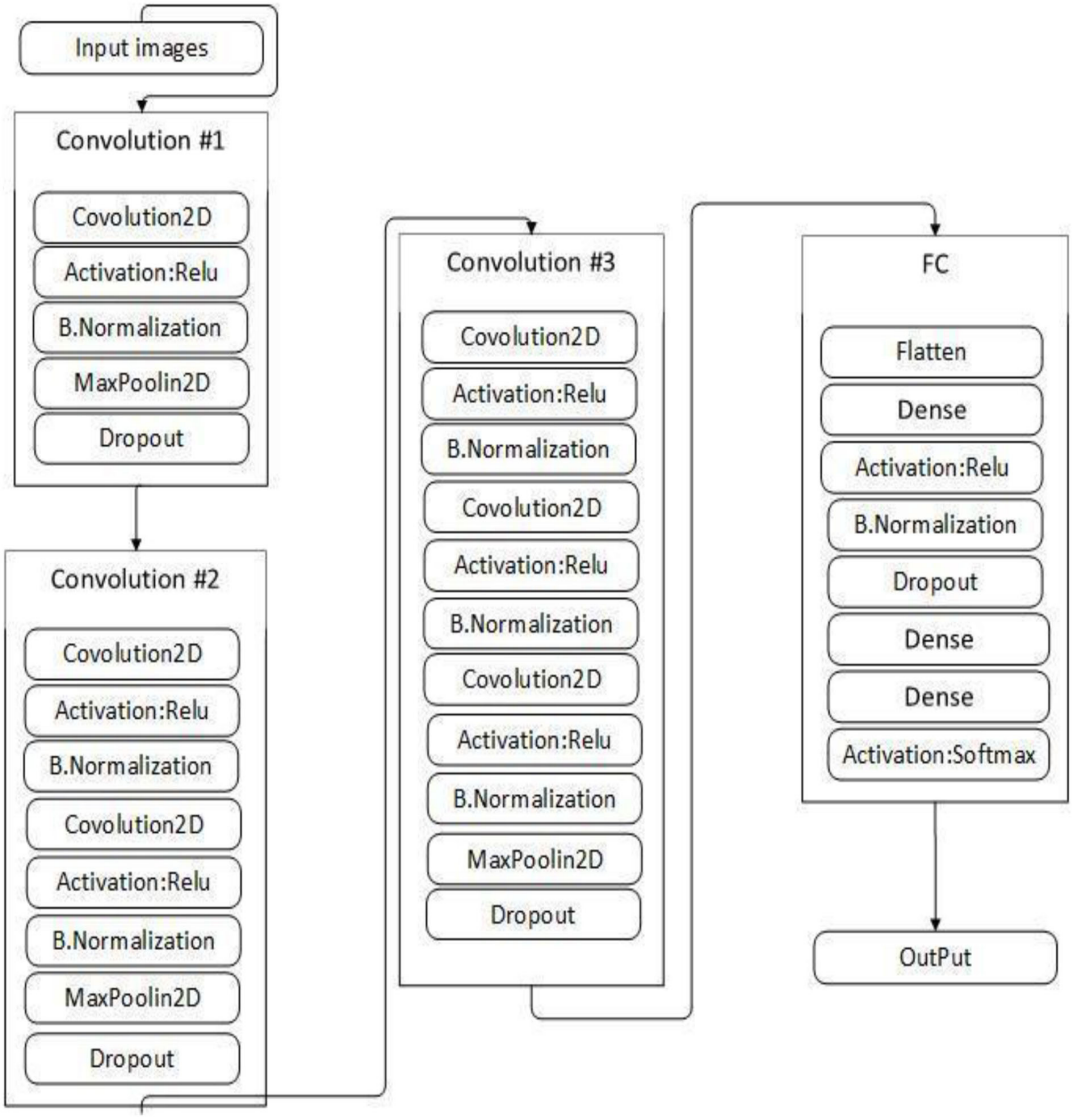
Proposed visual geometry group network (VGGNet) model architecture.

This study proposed VGGNet-based CNN architecture for breast cancer (BC) classification. The proposed model contained six convolutional layers, three max pooling, one flattening, and two fully connected layers. The detailed architecture of the proposed model is shown in Table 2 and Figure 9, while the pseudocode of the proposed VGGNet model is given in Algorithm 1.

**Table 2:** Proposed visual geometry group network (VGGNet) model architecture detail.

	**Convolution**	**Kernel**	**Padding**	**Stride**	**Input**	**Output**
1	Convolution	3 × 3	*P =* same	S = 1	(48,48,3)	(48,48,32)
2	ReLU	—–	—–	——	(48,48,32)	(48,48,32)
3	BNormalization	——–	———–	——-	(48,48,32)	(48,48,32)
4	Max pooling	3 × 3	*P =* same	S = 2	(48,48,32)	(24,24,32)
5	Dropout = 0.25	——–	———–	——–	(24,24,32)	(24,24,32)
6	Convolution	3 × 3	*P =* same	S = 1	(24,24,32)	(24,24,64)
7	ReLU	—–	—–	——	(24,24,64)	(24,24,64)
8	B Norml	——–	———–	——-	(24,24,64)	(24,24,64)
9	Convolution	3 × 3	*P =* same	S = 1	(24,24,64)	(24,24,64)
10	ReLU	—–	—–	——	(24,24,64)	(24,24,64)
11	Max pooling	3 × 3	*P =* same	S = 2	(24,24,64)	(12,12,64)
12	Dropout = 0.25	———	———–	——–	(12,12,64)	(12,12,64)
13	Convolution	3 × 3	*P =* same	S = 1	(12,12,64)	(12,12,128)
14	ReLU	—–	—–	——	(12,12,128)	(12,12,128)
15	B Normal	——–	———–	——-	(12,12,128)	(12,12,128)
16	Convolution	3 × 3	*P =* same	S = 1	(12,12,128)	(12,12,128)
17	ReLU	—–	—–	——	(12,12,128)	(12,12,128)
18	B Normal	——–	———–	——-	(12,12,128)	(12,12,128)
19	Convolution	3 × 3	*P =* same	S = 1	(12,12,128)	(12,12,128)
20	ReLU	—–	—–	——	(12,12,128)	(12,12,128)
21	Normalization	——–	———–	——-	(12,12,128)	(12,12,128)
22	Max pooling	3 × 3	*P =* same	S = 2	(12,12,128)	(6,6,128)
23	Dropout=0.25	——–	———–	——–	(6,6,128)	(6,6,128)
24	Flatten	——–	———-	——–	(6,6,128)	(4608)
25	Dense 1	——–	———–	——–	(4608)	(256)
26	ReLU	—–	—–	——	256	256
27	Normalization	——–	———–	——-	256	256
28	Dropout=0.25	——–	———–	——–	256	256
29	Dense 2	——–	———–	——–	256	2

**Algorithm 1:** Pseudocode of the proposed VGGNet model.

***Inputs:*** *Input the breast cancer images into the model*. ***Output**: display the output result images which have cancer* 1.***START*** 2. *Load training and testing data* 3. *Determine the total number of image paths in training* *and testing directories* 4. *Initialise proposed model and compile it with input* *dimensions, optimization function loss and evaluation metrics*. 5. ***for*** *I in number of Epochs* ***do*** 6. *Pass the input to the model with weights initialised randomly* 7. *The sensitivity of one layer is calculated from the previous* *layer and calculation of the update weights and bias and* *calculate loss, accuracy, precision, and recall and f-measure* 8. *Classify all the breast cancer images* 9. *Proposed VGGNet model keeps on calculating until all the* *data has been classified* 10.***end for*** 11.*Post process results and visualisation* 12.***END***

Table 2 presents the proposed architecture of a VGGNet-based CNN. The size of BC images is (48 × 48 × 3), which serves as an input to the VGGNet-based CNN. The 48 × 48 represents the height and width, and the 3 represents the dimensional channel. The proposed model consists of 12 layers in total. The convolutional layers are connected with the rectified linear unit (ReLU) activation function. The first fully connected layer is also connected with a ReLU activation function, while the final fully connected layer is connected with a SoftMax activation function. The final fully connected layer consists of two classes that classify the BC as either a malignant or benign tumor.

## Experimental results and discussion

Experiments were carried out several times with different architectures of deep learning algorithms. Various performance parameters, such as accuracy, recall, f-measure, precession, sensitivity, specificity, and loss, were used to evaluate the performance of the VGGNet-based CNN architecture. The dataset is divided into portions, each of which uses 70% for training and 30% for testing. The workstation used for the simulation is an Intel(R CPU) with 4 GB RAM running the Linux Ubuntu operating system using Keras, TensorFlow, application programming interface (API), matplotlib, NumPy, imutils, and OpenCV libraries. The simulation results of all three models are discussed in detail in the following section.

Table 3 illustrates the simulation results of the proposed VGGNet model as compared with the CNN and LeNet models in terms of accuracy and loss for training data. The simulation result of all three models is kept constant for up to 40 epochs. From the overall result, it shows that the proposed VGGNet shows a better performance than the other two models in terms of accuracy and loss. At the first epoch, the proposed model achieved an accuracy of 0.8412 with a loss of 0.441, while the accuracy of the CNN model starts at 0.7159% with a loss of 0.5537. Similarly, the LeNet model achieved an accuracy of up to 0.7523 with a loss of 0.4443. The result of all the models is recorded in every fifth iteration. Similarly, on 40 epochs, the proposed VGGNet model achieved an accuracy of up to 0.8638 with a loss of 0.3234. While the CNN and LeNet models achieved an accuracy of 0.7315, there was a loss of up to 0.7708 (0.4323, 0.3644).

**Table 3:** Performance analysis of convolutional neural network (CNN), LeNet, and proposed visual geometry group network (VGGNet) on training data.

**Iterations**	**CNN model**		**LeNet model**		**Proposed VGGNet model**	
	**Accuracy**	**Loss**	**Accuracy**	**Loss**	**Accuracy**	**Loss**
1	0.7159	0.5537	0.7523	0.4443	0.8412	0.4341
5	0.7188	0.5543	0.7522	0.4921	0.8402	0.4211
10	0.7191	0.5323	0.7564	0.4081	0.8586	0.3381
15	0.7120	0.5481	0.7544	0.3942	0.8547	0.3356
20	0.7256	0.5319	0.7685	0.3909	0.8599	0.3372
25	0.7308	0.4351	0.7533	0.3732	0.8719	0.3359
30	0.7319	0.4356	0.7464	0.3742	0.8621	0.3320
35	0.7321	0.4343	0.7656	0.3643	0.8631	0.3239
40	0.7315	0.4323	0.7708	0.3644	0.8638	0.3234

**Figure 10:**
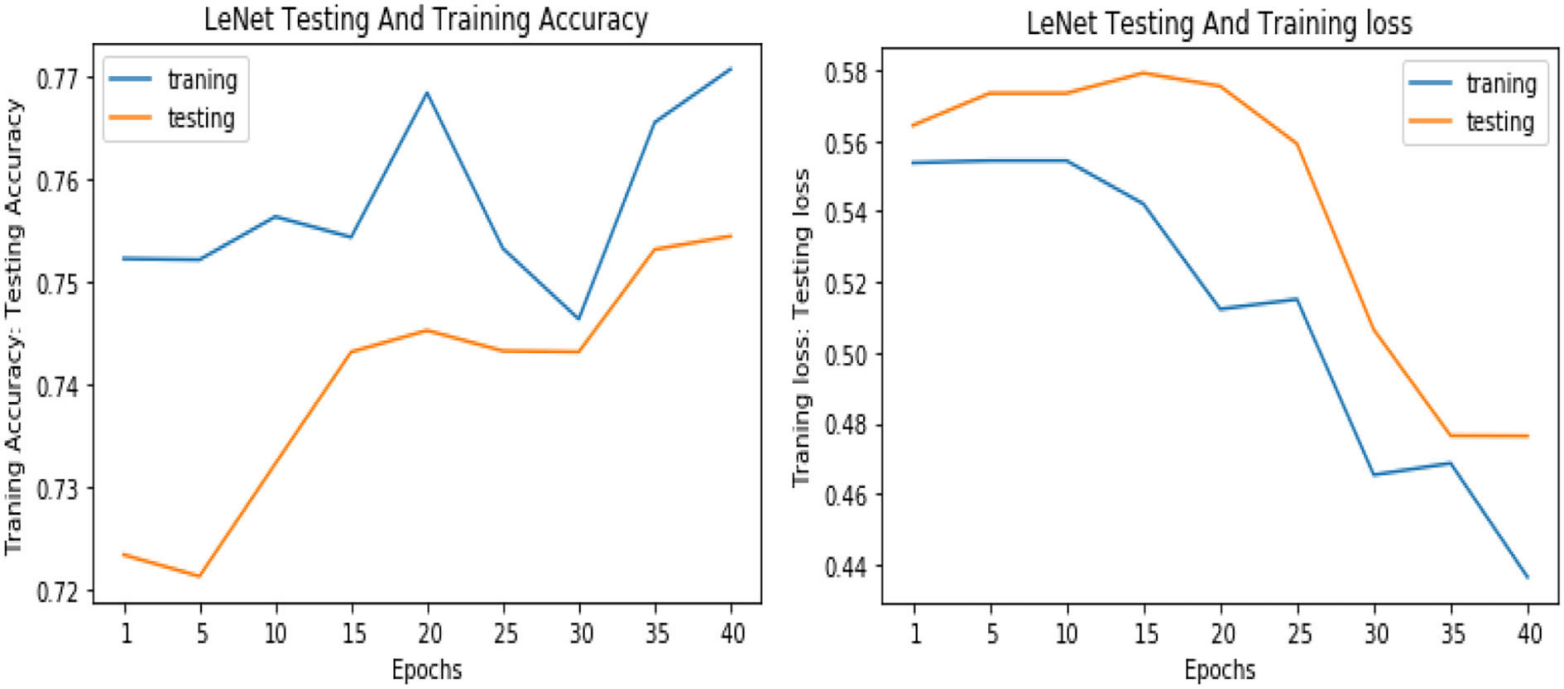
Accuracy and loss performance of LeNet model on training/testing data.

**Figure 11:**
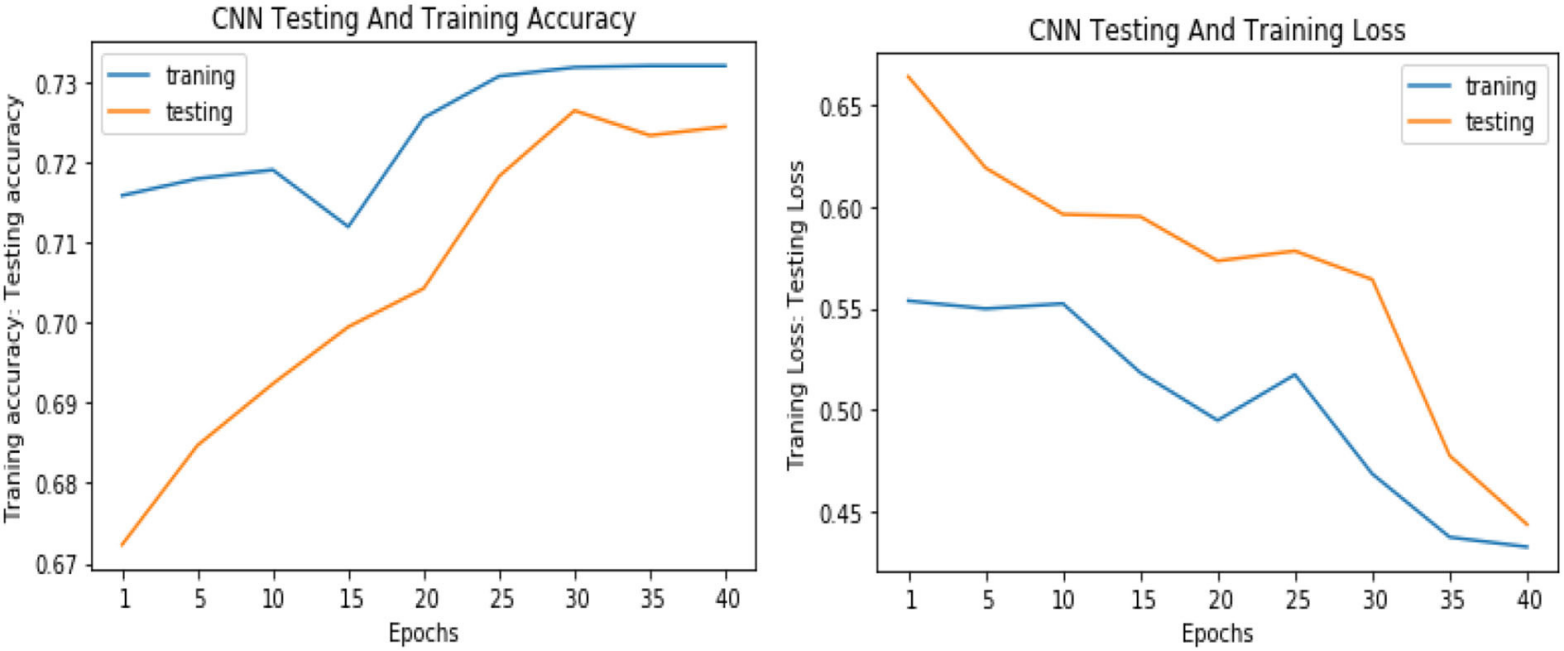
Accuracy and loss performance of the convolutional neural network (CNN) model on training/testing data.

**Figure 12:**
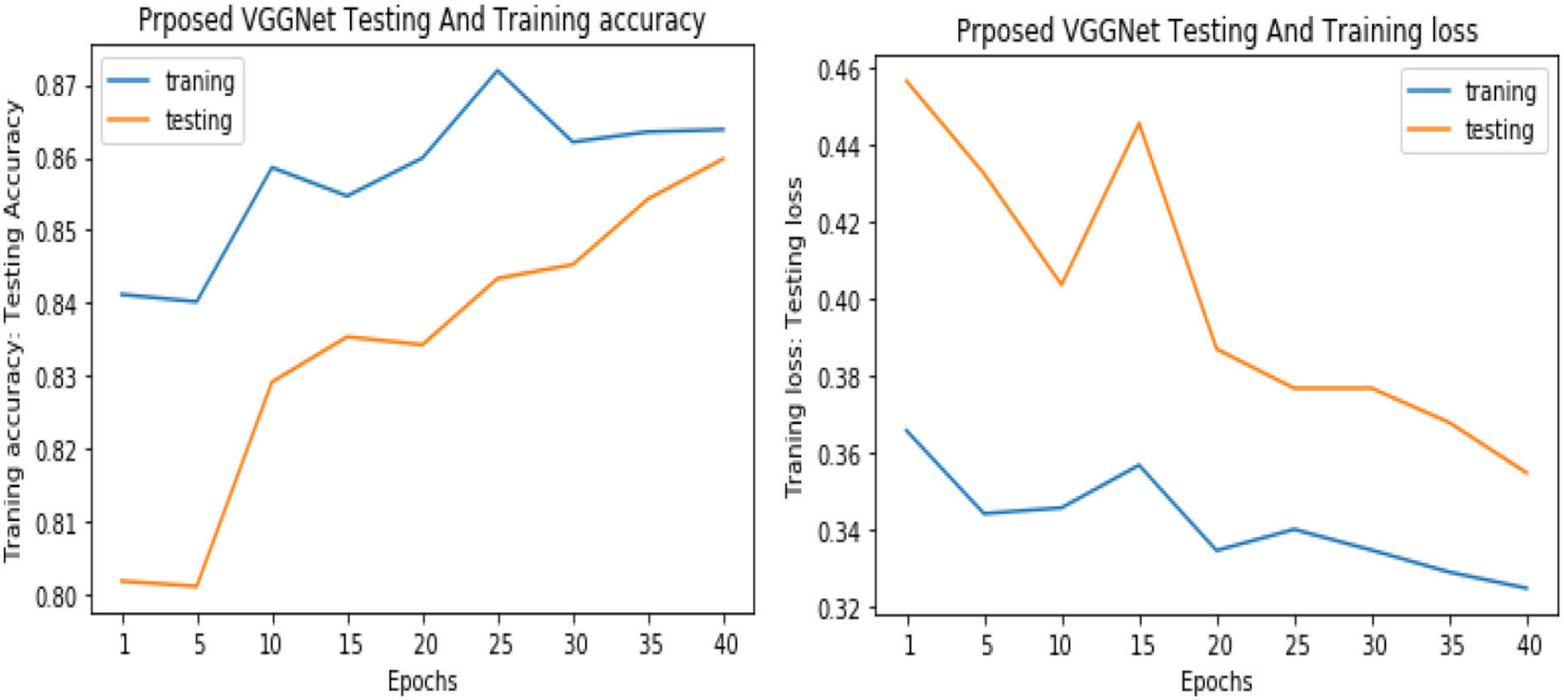
Accuracy and loss performance of proposed visual geometry group network (VGGNet) training/testing data.

Similarly, for the test data, the results of the proposed VGGNet, as compared with the CNN and LeNet models in terms of accuracy and loss, are presented in Table 4. For testing data, the results are recorded every fifth iteration. At the first iteration, the classical CNN achieved an accuracy of 0.6723 with a loss of 0.5643. The accuracy of the LeNet model starts at 0.7234 with a loss of 0.4232. Although the accuracy of the proposed VGGNet starts from 0.8019 with a loss of up to 0.4587, at iteration 5, the CNN achieved 0.6844% accuracy and the loss goes to 0.5434. While the LeNet reached 0.7213% accuracy and a loss of up to 0.4065. Furthermore, the proposed VGGNet model jumped to 0.8343% accuracy with a loss of up to 0.3876, which shows quite a better improvement in the result as compared to the other models in terms of accuracy and loss. Finally, after 40 iterations, the proposed model achieved 0.8598% accuracy with a loss of 0.3531. The accuracy of CNN and LeNet models reached 0.7265 and 0.7545 with a loss of up to 0.4120 and 0.3534, respectively, which is quite lower in performance than the proposed model in terms of accuracy and loss. Figure 10 shows the graphical representation of accuracy and loss convergence of the LeNet model both on the training and testing datasets, while the loss convergence and accuracy performance of the CNN model are given in Figure 11. The loss convergence and accuracy performance of the proposed model are represented in Figure 12.

**Table 4:** Performance analysis of convolutional neural network (CNN), LeNet, and proposed visual geometry group network (VGGNet) on testing data.

**Iterations**	**CNN model**		**LeNet model**		**Proposed VGGNet model**	
	**Accuracy**	**Loss**	**Accuracy**	**Loss**	**Accuracy**	**Loss**
1	0.6723	0.5643	0.8019	0.4587	0.7234	0.4232
5	0.6864	0.5434	0.8343	0.3876	0.7213	0.4065
10	0.6795	0.5423	0.8232	0.4420	0.7323	0.3876
15	0.6995	0.5342	0.8434	0.3769	0.7432	0.3644
20	0.6834	0.4254	0.8292	0.4243	0.7563	0.3797
25	0.71 85	0.4212	0.8453	0.3620	0.7433	0.3654
30	0.7065	0.4321	0.8354	0.3991	0.7532	0.3644
35	0.7182	0.4234	0.8543	0.3539	0.7532	0.3595
40	0.7265	0.4120	0.8598	0.3531	0.7545	0.3534

**Figure 13:**
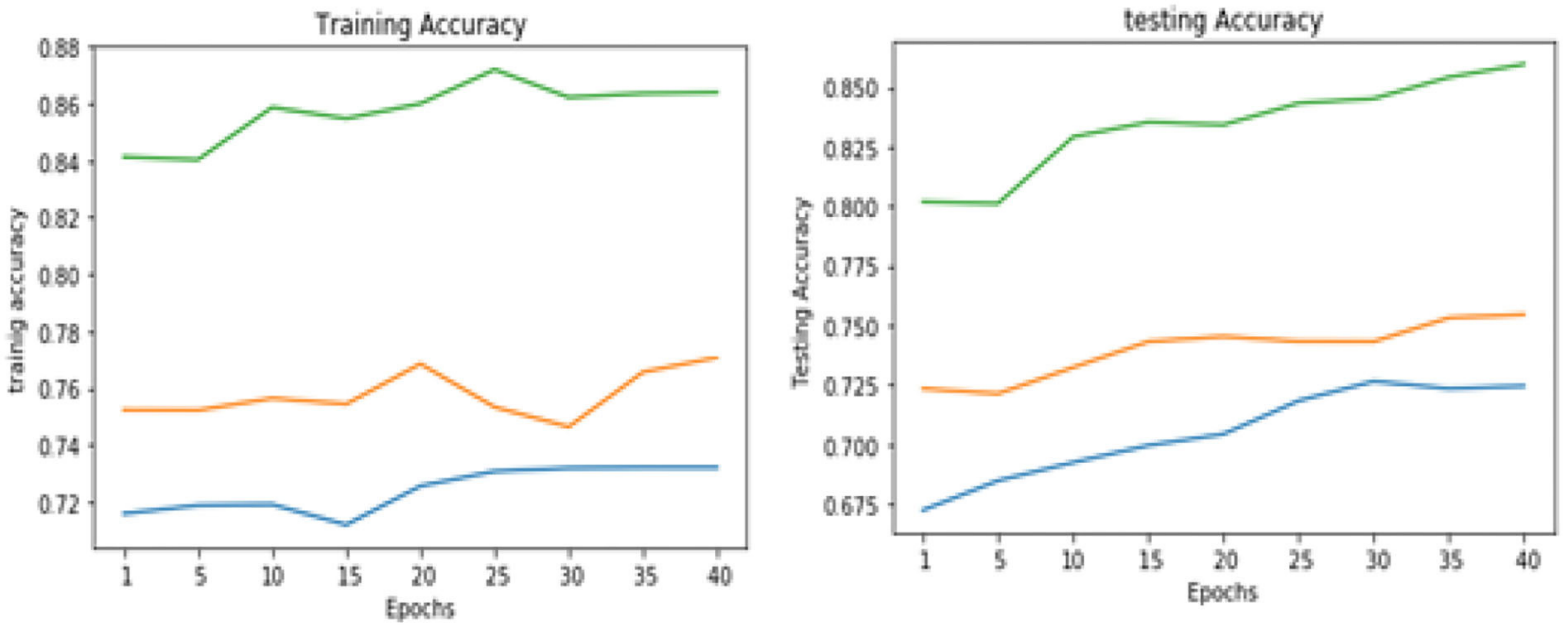
Training and testing accuracy comparison of three models.

**Figure 14:**
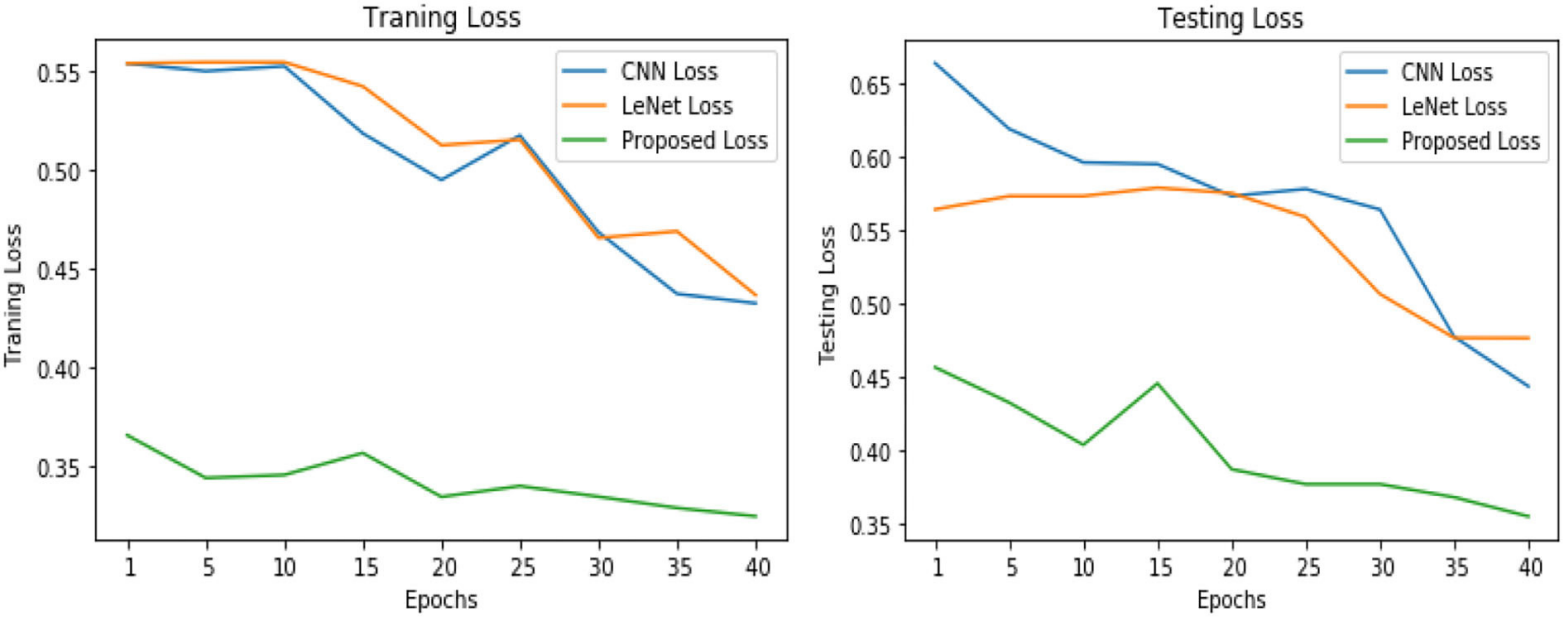
Training and testing loss comparison of three models.

Table 5 shows the overall performance of the proposed VGGNet as compared with conventional CNN and LeNet in terms of accuracy and loss, both for training and testing datasets. The table shows that the proposed model achieved 86.38% accuracy on training data, while 85.98% accuracy was attained on testing data with a loss of 0.3234 and 0.3531, respectively. While the LeNet fell behind the proposed model, which achieved 77.08% accuracy on training data with a loss of 0.3644 and achieved 75.45% accuracy on testing data with a loss of 0.4272. Similarly, the CNN model fell into the last position with an accuracy of 73.65% on training data, while 72.32% was obtained on testing data with a loss of 0.4398 and 0.4520, respectively. Table 5 clearly shows that the proposed VGGNet model attained high accuracy as compared to the other two models, because the proposed VGGNet in this study is the modified architecture of the CNN, which overcomes the overfitting issue in the existing model. Furthermore, Figures 13, 14 show the graphical representation of the accuracy and loss convergence of the three models, both for the training and testing datasets.

**Table 5:** Overall accuracy and loss comparison table of convolutional neural network (CNN), LeNet, and proposed visual geometry group network (VGGNet).

**Model**	**Accuracy**		**Loss**	
	**Training data**	**Testing data**	**Training data**	**Testing data**
VGGNet	86.38	85.98	0.3234	0.3531
LeNet	77.08	75.45	0.3644	0.4272
CNN	*73.65*	*72.32*	0.4398	0.4520

Finally, Table 6 shows the average evaluation performance of all three models in terms of precision recall f-measure sensitivity and specificity on testing data. Table 6 clearly shows that the proposed VGGNet outperformed both the LeNet and CNN. The proposed model achieved the precession, recall f-measure sensitivity, and specificity rate with 0.81, 0.85, 0.83, and 0.85 micro average and 0.85, 0.85, 0.85, 0.85, and 0.84 macro average. In this study, micro average means the sum of the individual true positive, true negative, and false positive results, while macro average means the average of the precision results. Similarly, the LeNet fell behind the proposed model with the precision, recall f-measure sensitivity, and specificity rate of 0.70, 0.74, and 0.72 micro average and 0.75, 0.77, 0.74, 0.71, and 0.67 of the macro average, where the CNN model achieved the precision, recall f-measure sensitivity, and specificity of 0.67, 0.70, 0.69, 0.74, and 0.71 of the macro average.

**Table 6:** Average evaluation performance of convolutional neural network (CNN), LeNet, and proposed visual geometry group network (VGGNet) on testing data.

**Model**	**Precision**		**Recall**		**F-Measure**		**Sensitivity**	**Specificity**
	**Micro-avg**	**Macro-avg**	**Micro-avg**	**Macro-avg**	**Micro-avg**	**Macro-avg**	**Avg**	**Avg**
VGGNet	0.81	0.85	0.85	0.85	0.83	0.85	0.85	0.84
LeNet	0.70	0.75	0.74	0.77	0.72	0.74	0.71	0.67
CNN	0.67	0.71	0.70	0.70	0.65	0.69	0.74	0.71

## Conclusion

This study presented a CNN architecture based on VGGNet for classifying breast cancer. Finding and identifying breast cancer is an extremely difficult process. In the present computer world, a variety of computational techniques like KNN, SVM, MLP, Decision Tree, and Genetic Algorithm were applied for the detection and classification of breast cancer. However, each approach has its own accuracy restrictions. As a result, our research suggested a new CNN built on the VGGNet. The CNN model's problem with classification accuracy can be fixed by the proposed model. The experimental findings in this study are based on three models, including CNN, LeNet, and suggested VGGNet models. The suggested model is demonstrated through simulation to be superior to the CNN and LeNet models in terms of accuracy, loss, f-measures, procession, and recall, whereas the CNN and LeNet models lag behind the proposed VGGNet based on the CNN model. In future, the experiment should be run using data from real breast cancer cases. Furthermore, the modified version of deep learning will be used to detect breast cancer in the images. Brain tumor, skin cancer, and different

kinds of other diseases will be detected by using YOLO (You Only Look Once) models.

## Data availability statement

The datasets presented in this study can be found in online repositories. The names of the repository/repositories and accession number(s) can be found below: The Breast Histopathology Images dataset used in the research work is available at: https://www.kaggle.com/paultimothymooney/breast-histopathology-images.

## Ethics statement

The studies involving human participants were reviewed and approved by Agriculture University, Peshawar. Written informed consent for participation was not required for this study in accordance with the national legislation and the institutional requirements.

## Author contributions

Conceptualization and validation: AbK, AsK, and MU. Formal analysis and software: AbK and MU. Funding acquisition and supervision: MA and MS. Investigation: AbK, AsK, and JB. Methodology: AbK, AsK, MU, and JB. Resources: AsK. Writing: AsK and AbK. Writing—review and editing: AbK and JB. All authors have read and agreed to the published version of the manuscript.

## Funding

The research work was partially sponsored and supported by the Faculty of Computing and Informatics at Multimedia University Malaysia.

## Conflict of interest

The authors declare that the research was conducted in the absence of any commercial or financial relationships that could be construed as a potential conflict of interest.

## Publisher's note

All claims expressed in this article are solely those of the authors and do not necessarily represent those of their affiliated organizations, or those of the publisher, the editors and the reviewers. Any product that may be evaluated in this article, or claim that may be made by its manufacturer, is not guaranteed or endorsed by the publisher.
